# Exocyst Subunits Exo70 and Exo84 Cooperate with Small GTPases to Regulate Behavior and Endocytic Trafficking in *C. elegans*

**DOI:** 10.1371/journal.pone.0032077

**Published:** 2012-02-28

**Authors:** Yaming Jiu, Congyu Jin, Yanbo Liu, Carina I. Holmberg, Jussi Jäntti

**Affiliations:** 1 Research Program in Cell and Molecular Biology, Institute of Biotechnology, University of Helsinki, Helsinki, Finland; 2 Research Programs Unit, Molecular Cancer Biology, and Institute of Biomedicine, University of Helsinki, Helsinki, Finland; BioScience Project, United States of America

## Abstract

The exocyst complex is required for cell polarity regulation and the targeting and tethering of transport vesicles to the plasma membrane. The complex is structurally well conserved, however, the functions of individual subunits and their regulation is poorly understood. Here we characterize the mutant phenotypes for the exocyst complex genes *exoc-7 (exo70)* and *exoc-8 (exo84)* in *Caenorhabditis elegans*. The mutants display pleiotropic behavior defects that resemble those observed in cilia mutants (slow growth, uncoordinated movement, defects in chemo-, mechano- and thermosensation). However, no obvious morphological defects in cilia were observed. A targeted RNAi screen for small GTPases identified eleven genes with enhanced phenotypes when combined with *exoc-7*, *exoc-8* single and *exoc-7;exoc-8* double mutants. The screen verified previously identified functional links between the exocyst complex and small GTPases and, in addition, identified several novel potential regulators of exocyst function. The *exoc-8* and *exoc-7;exoc-8* mutations caused a significant size increase in the *rab-10* RNAi-induced endocytic vacuoles in the intestinal epithelial cells. In addition, *exoc-8* and *exoc-7;exoc-8* mutations resulted in up-regulation of RAB-10 expression and affected the accumulation of endocytic marker proteins in these cells in response to *rab-10* RNAi. The findings identify novel, potential regulators for exocyst function and show that *exoc-7* and *exoc-8* are functionally linked to *rab-10* in endosomal trafficking in intestinal epithelial cells in *C. elegans*.

## Introduction

The exocyst is a functionally and structurally conserved multi-protein complex that is essential for cell polarity regulation in eukaryotic cells. It is involved in targeting and tethering of transport vesicles to the plasma membrane [1]–[4] and is composed of eight subunits, *sec3*, *sec5*, *sec6*, *sec8*, *sec10*, *sec15*, *exo70* (*exoc-7*) and *exo84* (*exoc-8*) [5], [6]. In yeast, loss-of-function mutations in exocyst subunits block protein secretion and lead to the accumulation of secretory vesicles [6], [7]. In mammalian epithelial cells the exocyst regulates membrane trafficking to the basolateral plasma membrane [8], [9] and regulates the localization of newly synthesized apical actin [10]. In addition, exocyst proteins have been linked to ciliogenesis of the primary cilia in mammalian cells [10], [11]. However, the functions of individual exocyst components and the mechanisms by which this tethering complex interact with other cell polarity components are poorly understood.

The small GTPases are key regulators of diverse cellular and developmental events, including differentiation, cell division, vesicle transport, nuclear assembly and control of the cytoskeleton [12]. In various model systems, the exocyst function has been shown to be regulated by a set of small GTPases [2], [13]. In yeast, the targeting and assembly of the exocyst complex is dynamically regulated by Sec4, Rho1, Rho3 and Cdc42 through distinct subunit interactions [5], [14]–[16]. Exo70p has been shown to interact with Cdc42 [17] and Rho3p [18] and the Rho3p-Exo70p interaction is important for efficient secretory function [19]. Unlike the interaction between Sec3p and Rho1p, which does not seemto be conserved for the mammalian exocyst complex, the Rho3p-Exo70p interaction is conserved, as the mammalian Exo70 binds the Rho family member TC10 [19]. The mammalian Exo84 and Sec5 are effectors of the Ral GTPases, RalA and RalB, which, however, are not found in yeast [20]–[23]. These results highlight the importance of small GTPases in exocyst function regulation and indicate that for some subunits variation in the molecular interactions and the modes of cooperation has occurred during evolution.

Here, using *C. elegans* as a model, we report that mutations in two exocyst subunits *exoc-7* and *exoc-8* result in behavioral phenotypes. Furthermore, we identify a set of small GTPases by RNAi screening that are functionally linked to *exoc-7* and *exoc-8*. The phenotypes induced by RNAi of *rab-10* (one of the genes identified in the screen) in *exoc-7*, *exoc-8* and *exoc-7;exoc-8* mutants suggest that RAB-10, EXOC-7 and EXOC-8 cooperate in membrane recycling from the endosomal compartment to the plasma membrane in intestinal epithelial cells in *C. elegans*.

## Results

### Exocyst subunit *exoc-7* and *sec-6* are broadly expressed during *C. elegans* development

**Figure 1:**
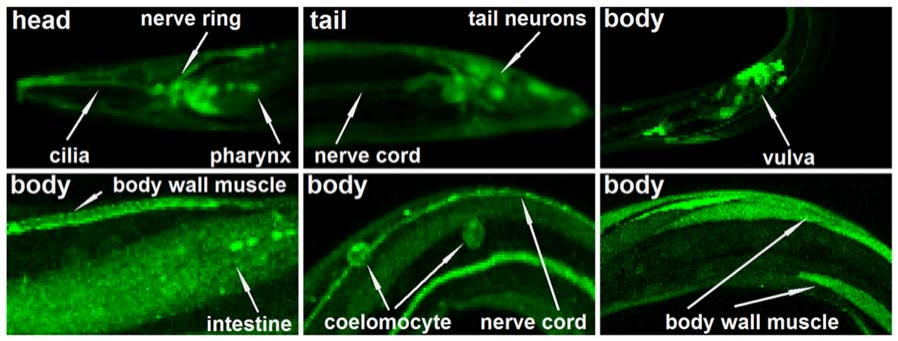
Expression patterns of exocyst subunit *exoc-7* in *C. elegans*. Confocal images of adult hermaphrodites expressing P*exoc-7*::GFP under the 2954 bp promoter (Ex[P*exoc-7*::GFP; *pRF4*]).

The functional role of the exocyst complex in the development of *C. elegans* is largely uncharacterized. In order to evaluate the expression pattern of exocyst subunits, transgenic animals expressing transcriptional GFP reporters were generated for two previously uncharacterized exocyst subunit genes in *C. elegans*. The expression of P*exoc-7*::GFP was observed from embryo to adulthood (data not shown), through all developmental stages. In adults, strong expression was observed in multiple tissues including nerve ring, pharynx, tail neurons, dorsal and ventral cord, coelomocyte and intestine as well as in vulva, seam cells and body wall muscle (Figure 1). Similarly to *exoc-7*, strong expression of *sec-6* was observed in the nervous system including nerve ring and nerve cord in adult animals, while weaker expression was seen in other tissues, such as intestine and muscle (Figure S1). These broad expression patterns are consistent with the reported expression pattern of the transcriptional exocyst subunit reporter P*sec-5*::GFP [24] and are well in line with the anticipated role of exocyst complex and its subunits as important regulators of cell polarity.

### *exoc-7*, *exoc-8* and *exoc-7;exoc-8* mutant worms display pleiotropic behavior defects

The *C. elegans exoc-7* (*ok2006*) and *exoc-8* (*ok2523*) mutant animals are viable (eight times back-crossed to wild type N2 animals). The *ok2006* allele of *exoc-7* has a 1803 bp deletion and the *ok2523* allele of *exoc-8* has a 1474 bp deletion (Figure S2A). In the wild type animals, RT-PCR analysis with two oligonucleotide pairs verified expression of an mRNA for *exoc-7*. However, in the *exoc-8* mutant animals no detectable transcript is produced (Figure S2A and B). When RNAi bacteria targeting *exoc-7* and *exoc-8* were fed to *exoc-7* and *exoc-8* mutant worms, respectively, no additional phenotypes were observed (data not shown). The data suggest that *exoc-8(ok2523)* is a null mutation. However, based on the RT-PCR analysis the *exoc-7(ok2006)* cannot be conclusively defined as a null mutant.

**Figure 2:**
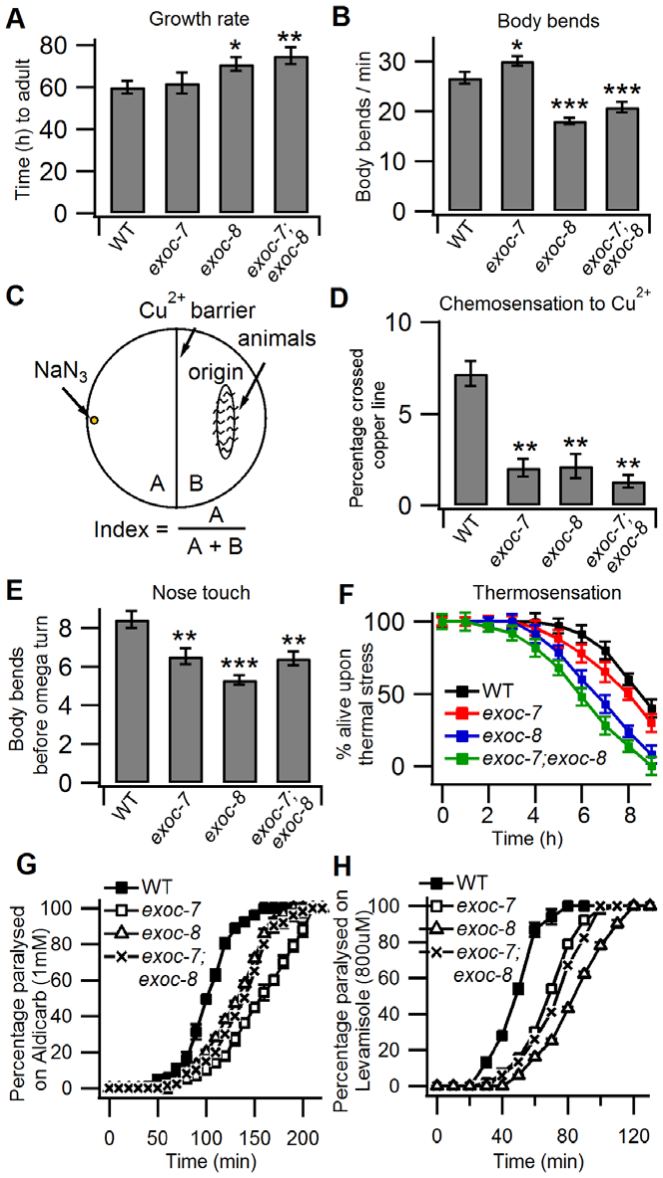
Characterization of the behavioral phenotypes in *exoc-7*, *exoc-8* single and *exoc-7;exoc-8* double mutant worms. (A) Quantification of the growth rate to adulthood in wild type (n = 15), *exoc-7* (n = 18), *exoc-8* (n = 23) and *exoc-7;exoc-8* (n = 16) worms. (B) Quantification of wild type (n = 35), *exoc-7* (n = 42), *exoc-8* (n = 33) and *exoc-7;exoc-8* (n = 39) animal locomotion defect. (C) A schematic presentation of the experimental setup used in the Cu^2+^ avoidance assay. NaN_3_ was spotted on one side of the plate divided by a 100 mM Cu^2+^ (or Cd^2+^) barrier spread on the midline of the plate. For each experiment 200-400 washed adult worms were placed on the opposite side [B] to the NaN_3_ and their ability to traverse the Cu^2+^ to the other side [A] was scored. The index was calculated as A/(A+B). (D) Quantification of the Cu^2+^-sensitivity for wild type, *exoc-7, exoc-8* and *exoc-7;exoc-8* worms. (E) Quantification of the sensitivity to gentle nose touch for wild type (n = 25), *exoc-7* (n = 21), *exoc-8* (n = 27) and *exoc-7;exoc-8* (n = 29) worms. (F) The effect of thermal shock (35°C) on viability of wild type, *exoc-7*, *exoc-8* and *exoc-7;exoc-8* worms. (G) and (H) Analysis the resistance to aldicarb and levamisole in wild type, *exoc-7*, *exoc-8* and *exoc-7;exoc-8* worms. Asterisks denote statistical significance as compared to controls, with a P value less than 0.05 (*), 0.01 (**) and 0.001 (***).

In order to explore the interplay of EXOC-7 and EXOC-8 in the function of the exocyst complex, *exoc-7;exoc-8* double mutant animals were generated and possible phenotypes were analyzed. When the growth rate from newly laid eggs to adults was investigated in *exoc-7*, *exoc-8* single and *exoc-7;exoc-8* double mutants, a mild defect was observed for *exoc-8* and *exoc-7;exoc-8* double mutants (Figure 2A). Analysis of the mutant animal movement revealed that compared to wild type worms, *exoc-7* mutant animals showed a slight increase in the number of body bends per minute, while *exoc-8* and *exoc-7;exoc-8* mutants showed decreased locomotion (Figure 2B).

Abnormal movement can indicate compromised neuronal function. Therefore, different assays for behavior and sensing were utilized. First, wild type and mutant animals were subjected to an assay for Cu^2+^-sensing (Figure 2C). When a population of wild type worms was placed on a petri dish, they dispersed evenly within a few minutes. However, when a Cu^2+^ line was placed in the middle of the plate, only a few animals crossed the Cu^2+^ line and reached the other side of the plate. When the ability of *exoc-7* and *exoc-8* single and *exoc-7;exoc-8* double mutants to cross the Cu^2+^ line was compared to that of wild type animals, all mutants were less likely to cross the 100 mM Cu^2+^ barrier (Figure 2D). A similar defect was observed at lower concentrations of Cu^2+^ (10 mM, 50 mM) (Figure S3B) and another repulsive ion Cd^2+^ at 100 mM, (Figure S3A). To exclude the possibility that the mildly compromized movement of *exoc* mutants affect the behavior of animals in the test, the *unc-10(e102)* mutant animals were used as a negative control. The *unc-10* gene encodes a presynaptic protein that binds and affects the activity of synaptic vesicles. *unc-10* animals displayed a more severe locomotion defect than *exoc* mutants (Figure S3C). However, they displayed similar Cu^2+^ sensitivity as the wild type animals (Figure S3D). This suggests that the Cu^2+^ hypersensitivity phenotype of *exoc* mutants is not caused by fewer body bends or reduced mobility. The *exoc-7*, *exoc-8* single and *exoc-7;exoc-8* double mutations resulted in a slightly reduced response to nose touch, suggesting a mild defect in mechanosensation (Figure 2E). The *exoc-8* and *exoc-7;exoc-8* double mutant animals were more sensitive to thermal stress than *exoc-7* single mutant or wild type animals (Figure 2F). However, compared to wild type animals, *exoc-7*, *exoc-8* single and *exoc-7;exoc-8* double mutants had similar sensitivity to a high osmotic glycerol circle (data not shown). Furthermore, no differences were observed in pharyngeal pumping rate, brood size and life span (Table S1). In a more direct analysis for neuronal function, both *exoc-7, exoc-8* single and *exoc-7;exoc-8* double mutants displayed insensitivity to the acetylcholinesterase inhibitor aldicarb and acetylcholine receptor agonist levamisole (Figure 2G and 2H). This suggests defects in the endogenous acetylcholine release to neuromuscular junctions or decreased amount/dysfunction of nicotinic acetylcholine receptors in these mutants.

The observed phenotypes described above resemble those typically observed for ciliary mutants [25]. However, in contrast to a previously characterized *che-3(e1124)* dynein heavy chain mutant [26], no obvious morphological defects were observed in *exoc* mutants in cilia structures using the DiI staining (Figure S4). Similarly, no obvious morphological defects were observed in the Cu^2+^-sensing ASEL/R, ADL, ASH neurons and temperature-sensing AFD neuron marked by GFP expression from neuronal specific promoters of *gcy-5/7*, *gpa-15* and *gcy-8*, respectively (Figure S4). Furthermore, cilia length and the fluorescence intensity of ASEL/R, ADL and AFD neurons appeared identical to those of the wild type animals (data not shown). This suggests that the behavioral phenotypes observed caused by *exoc* mutant induced defects on neuronal cell function(s) rather than on the morphology of the neurons.

### *rab-8* and *ral-1* regulate exocyst function in *C. elegans*

**Figure 3:**
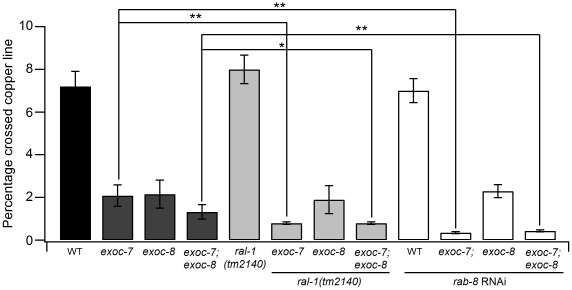
*ral-1* and *rab-8* display differential genetic interactions with *exoc-7*, *exoc-8* and *exoc-7;exoc-8* in the Cu^2+^-sensing assay. The analysis for Cu^2+^ avoidance in *ral-1*, *rab-8* RNAi, *exoc-7*, *exoc-8* and *exoc-7;exoc-8* mutants and their different combinations. The experimental setup was identical to that described in Figure 2C. Asterisks denote statistical significance as compared to controls, with a P value less than 0.05 (*), 0.01 (**) and 0.001 (***).

**Figure 4:**
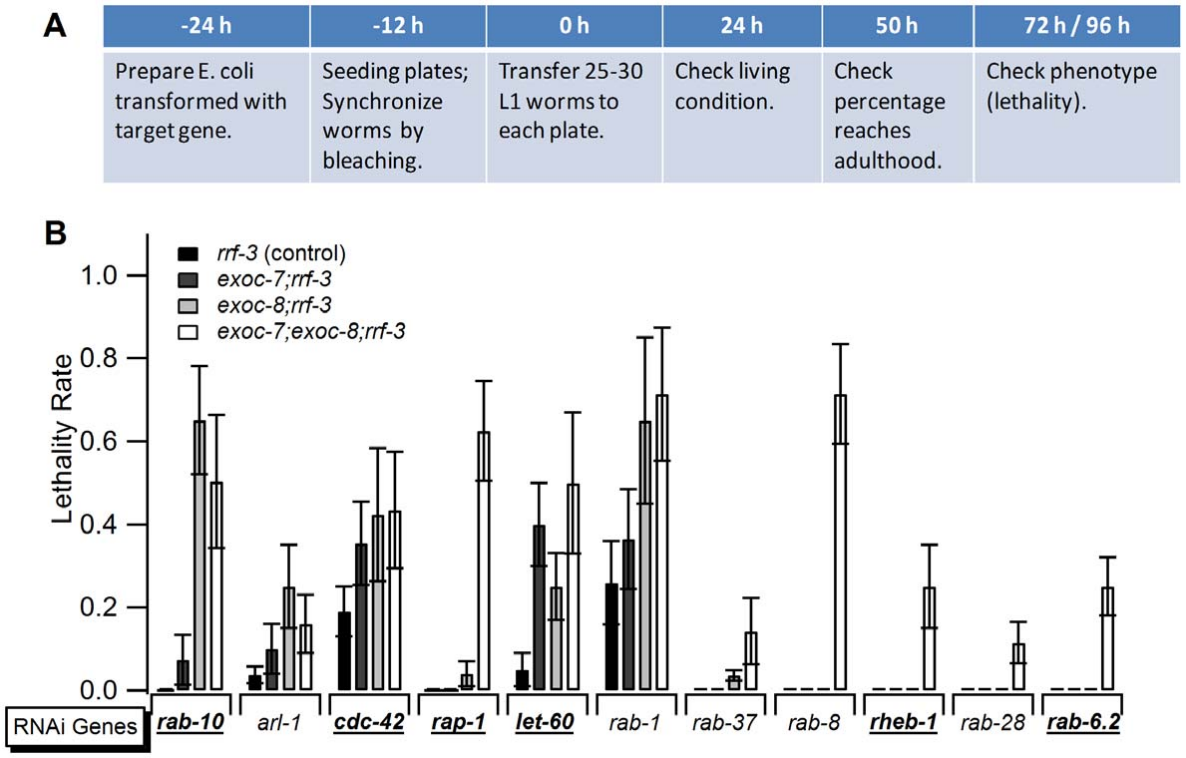
An RNA-mediated interference screen for small GTPases reveals novel GTPases functionally linked to *exoc-7* and *exoc-8*. (A) The design of the screen. (B) The synthetic lethality rates of the candidates identified in the screen. Underlining/bold indicates genes where the enhanced phenotypes were also observed in the wild type N2 background.

Small GTPases *ral-1* and *rab-8* have been reported to regulate the function of the exocyst complex in mammalian cells [27]–[29]. In mammalian cells Sec5 and Exo84 (Exoc8) act as effectors for RalA, whereas Rab8, the mammalian homologue of yeast Sec4p, has been implicated in exocyst regulation through interactions with Sec15p [15], [22]. When compared to wild type animals, no defects in the Cu^2+^-sensing assay were observed for *ral-1(tm2140)* mutant and *rab-8* RNAi treated worms (Figure 3). However, the Cu^2+^ avoidance was enhanced when this *ral-1* mutation or RNAi treatment for *rab-8* were combined with *exoc-7* and *exoc-7;exoc-8* mutations. At the same time, no detectable combined effect for this phenotype was observed for *exoc-8* mutation (Figure 3). In the RNAi-sensitive *rrf-3* background, the *rab-8* RNAi results in synthetic lethality in *exoc-7;exoc-8* double mutant worms (Figure 4). In line with this, an additive effect for the combination of *exoc-7* and *exoc-7;exoc-8* with *rab-8* RNAi was observed when the Cu^2+^-sensing assay was carried out in the wild type N2 background (Figure 3). These results suggest that defects in *ral-1* and *rab-8* affect *exoc-7* and *exoc-*8 function differentially.

### An RNAi screen for small GTPases in *exoc-7*, *exoc-8* and *exoc-7;exoc-8* mutant worms reveals novel, potential exocyst regulators

To gain a deeper understanding on the role of small GTPases in exocyst complex regulation, an RNAi screen was carried out that scored for enhanced growth defects for *exoc-7*, *exoc-8* single and *exoc-7;exoc-8* double mutants in response to down-regulation of the small GTPase genes encoded in the *C. elegans* genome. The screen was performed by transferring synchronized L1 worms to RNAi plates and observing phenotypes three days thereafter (Figure 4A). Eleven candidate genes were identified with a synthetic lethal growth phenotype when combined with *exoc-7*, *exoc-8* and *exoc-7;exoc-8* mutants in the *rrf-3* background (Figure 4B, Table S2).

All of the genes identified are highly conserved in metazoans. Some of the candidates identified in the RNAi screen (*rab-10* and *arl-1*) formed a more severe combination with the single *exoc-8* mutation than with the *exoc-7;exoc-8* double mutation. However, typically, the most severe phenotypes were observed when RNAi was performed in *exoc-7;exoc-8* double mutants (Figure 4B). The screen identified *rab-8*, *rab-10*, *rap-1* and *cdc-42* that have been previously shown to be functionally linked to the exocyst [1], [13], [17]. RAB-8, RAB-10, CDC-42, ARL-1 and LET-60 proteins are well known for their importance in the regulation of cell polarity, intracellular membrane trafficking, vulval development and cilia generation. Importantly, several genes were identified that have not previously been implicated in exocyst function regulation. These include RHEB-1, RAB-37, RAB-28 and RAB-6.2 (Figure 4B). In the future, additional work will be required to prove a functional link between these and the exocyst. The positive hits *rab-10*, *cdc-42*, *rap-1*, *let-60*, *rheb-1* and *rab-6.2* could also be verified in the wild type N2 background, while the rest, including *arl-1*, *rab-1*, *rab-37*, *rab-8*, and *rab-28*, were only observed in the RNAi-sensitive *rrf-3* background [30]. The attempts to combine *rab-10(dx-2)* mutation with the *exoc-7(ok2006)* and *exoc-8(ok2523)* mutations did not yield viable homozygous animals supporting a functional link between *rab-10* and the exocyst in *C. elegans*.

To verify the efficiency of RNAi in our screen, qRT-PCR was used to quantify the knock down efficiency of the candidate genes. After RNAi, *rab-8*, *rab-10* and *rheb-1* mRNA levels were decreased by 81%, 90% and 77%, respectively (Figure S5). It is worth noticing that knock down of one candidate seems to affect the level of others. For example, knock down of *rab-8* caused a compensatory increase in *rab-10* expression, and *vice versa*.

Compared to wild type animals, the *exoc-7*, *exoc-8* and *exoc-7;exoc-8* mutants displayed enhanced sensitivity to Cu^2+^ (Figure 2C). Therefore, the same test was performed for all of the eleven genes identified in the screen. The *rab-10*, *cdc-42*, and *rab-1* RNAi caused a strong locomotion defect or partial lethality. They were therefore not suitable for chemosensation assays. However, knock down of *let-60* resulted in insensitivity to Cu^2+^ and *arl-1*, *rheb-1* and *rab-37* RNAi worms behaved similarly in response to Cu^2+^ as did *exoc-7*, *exoc-8* and *exoc7;exoc-8* double mutants, respectively (Figure S6). This suggests that in addition to cooperating with the exocyst in growth regulation, these three small GTPase are also directly or indirectly involved in chemosensation regulation in *C. elegans*.

### *exoc-7*, *exoc-8* and *exoc-7;exoc-8* mutants affect RAB-10 expression and the size of endocytic vacuoles induced by *rab-10* down-regulation

**Figure 5:**
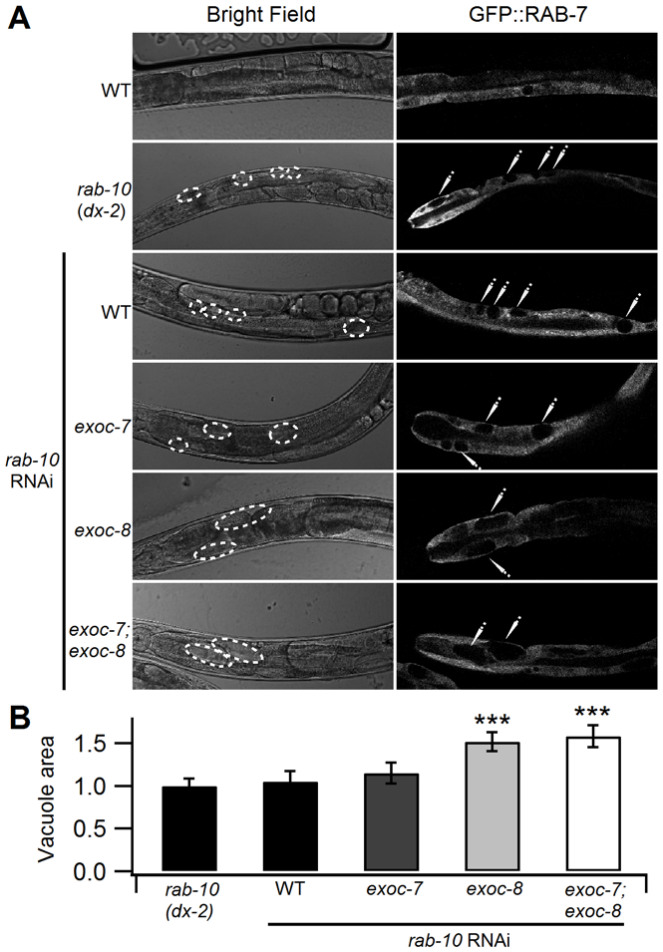
*exoc-8* and *exoc-7;exoc-8* mutations induce the formation of enlarged vacuoles in the intestinal epithelial cells treated with *rab-10* RNAi. (A) Representative images in bright field (left) and corresponding fluorescent images of GFP::RAB-7 (right) in the intestinal cells show enlarged vacuoles in *exoc-8* and *exoc-7;exoc-8* mutant background. (B) The quantification of the average vacuolar areas in different mutants (for *rab-10*(*dx-2*) n = 26, for WT treated *rab-10* RNAi n = 27, for *exoc-7* treated *rab-10* RNAi n = 25, for *exoc-8* treated *rab-10* RNAi n = 31, for *exoc-7;exoc-8* treated *rab-10* RNAi n = 28). Asterisks denote statistical significance as compared to controls, with a P value less than 0.05 (*), 0.01 (**) and 0.001 (***).

*C. elegans* intestine is composed of one layer of polarized epithelial cells [31]. The apical microvillar surface faces the lumen and is responsible for nutrient uptake from the environment. The basolateral surface faces the pseudocoelom (body cavity) and is responsible for the exchange of molecules between the intestine and the rest of the body [32]. It has been shown that *rab-10* mutant worms have large vacuoles in the intestine and that RAB-10 is required for basolateral endocytic recycling [33]. Recent data suggest that Rab10 may be co-operating with the exocyst in mammalian epithelial cells [34]. We therefore tested whether such cooperation would also exist in *C. elegans* and what consequences such cooperation might have. The *exoc-7*, *exoc-8* and *exoc-7;exoc-8* mutants have a morphologically normal intestine (data not shown). However, *exoc-8* single and *exoc-7;exoc-8* double mutations combined with *rab-10* RNAi resulted in clear growth of the *rab-10* RNAi induced GFP::RAB-7-positive vacuoles in size in the intestinal cells (Figure 5). At the same time, the number of vacuoles did not change (Data not shown).

**Figure 6:**
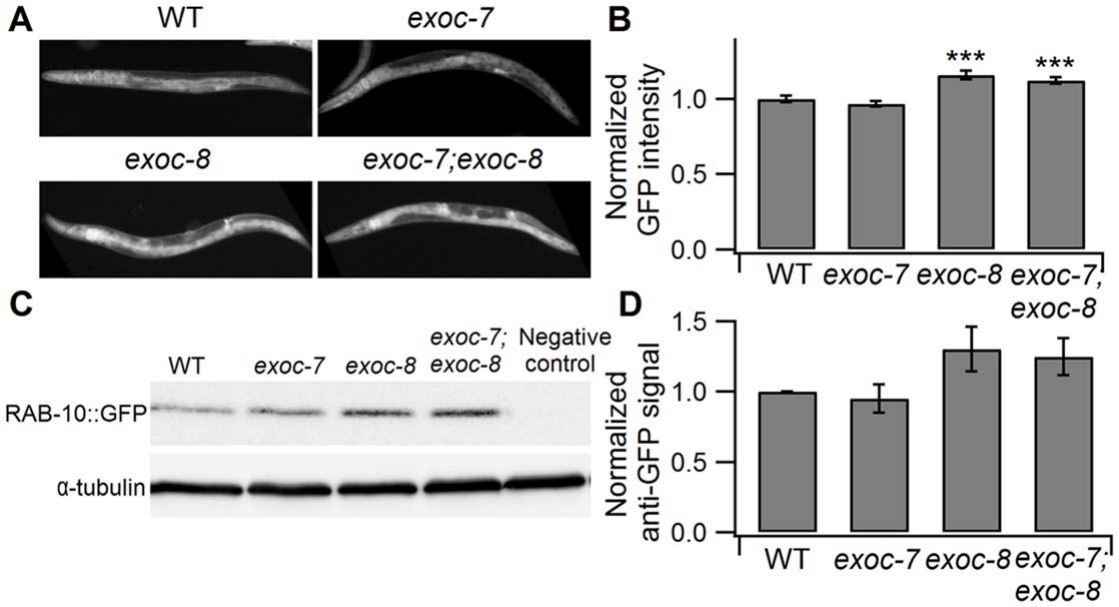
*exoc-8* and *exoc-7;exoc-8* mutations result in up-regulation of RAB-10 protein expression. (A) GFP::RAB-10 fluorescence is increased in *exoc-8* and *exoc-7;exoc-8* mutants. (B) Normalized average intensity of GFP fluorescence in transgenic strains Is[*rab-10*::GFP::RAB-10] of wild type (n = 36), *exoc-7* (n = 43), *exoc-8* (n = 47) and *exoc-7;exoc-8* (n = 40) mutant background. (C) A representative Western blot for GFP::RAB-10 in WT, *exoc-7*, *exoc-8* and *exoc-7;exoc-8* mutants. (D) Quantification of the GFP::RAB-10 Western blot result from four independent experiments. α-tubulin was used for normalization. Asterisks denote statistical significance as compared to controls, with a P value less than 0.05 (*), 0.01 (**) and 0.001 (***).

RAB-10 and exocyst subunits share a broad expression profile and appear functionally interlinked [33], [34]. The effect of *exoc-7* and *exoc-8* mutations on RAB-10 expression was tested. RAB-10 has been localized to early endosomes and Golgi in both neuron and intestine [33], [35]. When RAB-10 localization was analyzed in *exoc-7*, *exoc-8* single and *exoc-7;exoc-8* double mutants, no detectable difference was observed compared to the localization in the wild type animals (data not shown). However, compared to wild type and *exoc-7* mutant animals, a stronger fluorescence signal for GFP::RAB-10 (expression driven by its own promoter) was observed in *exoc-8* and *exoc-7;exoc-8* mutants (Figure 6A and 6B). The observed increase in GFP::RAB-10 expression was confirmed by Western blotting. As shown in Figure 6C, increased levels of GFP::RAB-10 was detected in *exoc-8* and *exoc-7;exoc-8* mutants, but not in *exoc-7* mutant animals. Taken together, these results indicate that *exoc-8* and *exoc-7;exoc-8* mutations affect the level of RAB-10 expression and suggest the existence of a compensation mechanism to increase RAB-10 expression in *exoc-8* and *exoc-7;exoc-8* mutant worms.

### *exoc-7* and *exoc-8* are functionally linked to *rab-10* in vesicular transport regulation in *C. elegans* intestine

RAB-10 is implicated in membrane recycling from early endosomes back to the plasma membrane in mammalian cells. The targeting of the recycling elements to the plasma membrane is likely to be regulated by the exocyst. In order to in a more direct way test the possible functional link between RAB-10 and the exocyst complex, the recycling of different marker proteins from the endosomal compartment to the plasma membrane was assessed in *exoc-7*, *exoc-8* single and *exoc-7*; *exoc-8* double mutants.

**Figure 7:**
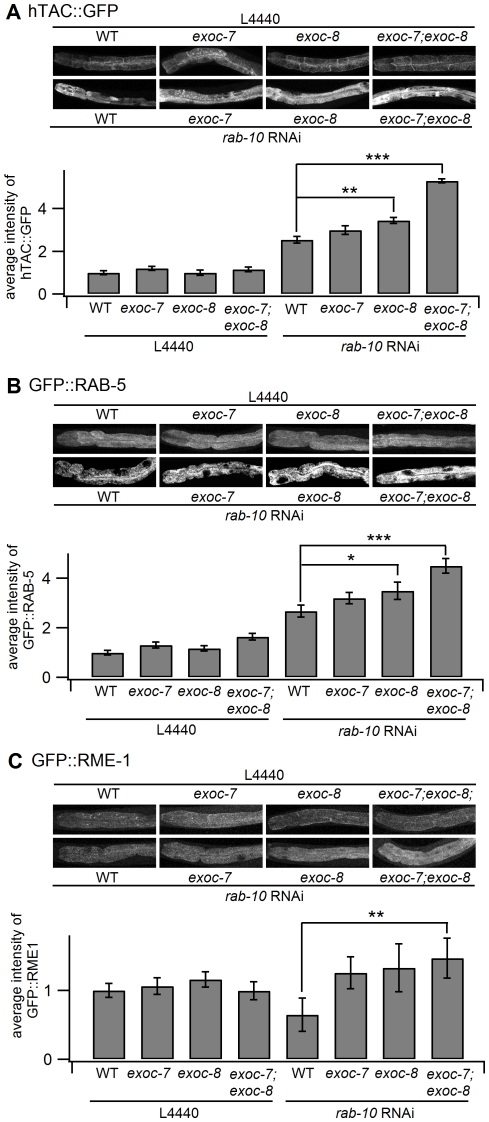
*exoc-7*, *exoc-8* single and *exoc-7;exoc-8* double mutations affect the *rab-10* RNAi-induced endocytic phenotype. (A) *rab-10* RNAi causes enhanced accumulation of the trans-membrane protein hTAC in *exoc-8* and *exoc-7;exoc-8* double mutants (WT n = 37, *exoc-7* n = 29, *exoc-8* n = 30, *exoc-7;exoc-8* n = 31, WT treated *rab-10* RNAi n = 27, *exoc-7* treated *rab-10* RNAi n = 32, *exoc-8* treated *rab-10* RNAi n = 38, *exoc-7;exoc-8* treated *rab-10* RNAi n = 36). (B) Early endosome marker GFP::RAB-5 is accumulated in *exoc-8* and *exoc-7;exoc-8* double mutants subjected to *rab-10* RNAi (WT n = 27, *exoc-7* n = 29, *exoc-8* n = 30, *exoc-7;exoc-8* n = 29, WT treated *rab-10* RNAi n = 27, *exoc-7* treated *rab-10* RNAi n = 25, *exoc-8* treated *rab-10* RNAi n = 33, *exoc-7;exoc-8* treated *rab-10* RNAi n = 28). (C) The accumulation of GFP::RME-1 in *rab-10* RNAi-treated *exoc-7*, *exoc-8* and *exoc-7;exoc-8* double mutant animals (WT n = 27, *exoc-7* n = 22, *exoc-8* n = 30, *exoc-7;exoc-8* n = 27, WT treated *rab-10* RNAi n = 23, *exoc-7* treated *rab-10* RNAi n = 26, *exoc-8* treated *rab-10* RNAi n = 31, *exoc-7;exoc-8* treated *rab-10* RNAi n = 29). In each sub-figure the upper panels show representative images of the GFP tagged protein in intestinal cells. The lower panels show the quantification of the normalized average fluorescence intensity. Asterisks denote statistical significance as compared to controls, with a P value less than 0.05 (*), 0.01 (**) and 0.001 (***).

The hTAC is the α-chain of the human IL-2 receptor TAC, a marker for clathrin-independent endocytosis and the Eps15-homology (EH)-domain protein RME-1-dependent recycling. The hTfR is the human transferrin receptor, a marker for clathrin-dependent endocytosis and RME-1-dependent recycling in mammalian cells [33]. These marker proteins have been previously used in *C. elegans* to study transport within the endocytic pathway [33]. The clathrin-dependent and clathrin-independent cargo are likely to meet in the endosomal system and RAB-10 has been proposed to regulate endocytic recycling, but not endocytosis *per se* [36]. Both *exoc-8* and *exoc-7;exoc-8* mutations enhanced the accumulation of hTAC in the intestine of *rab-10* RNAi treated worms (Figure 7A). The accumulation of hTAC suggests that there is a blockage in the endocytic recycling pathway that causes hTAC to accumulate inside the cell. However, no increase in the accumulation of hTfR was seen in *exoc-7* and *exoc-8* worms in response to *rab-10* RNAi (Figure S7A). These results suggest that hTAC recycling is sensitive for defects in *exoc-8* single and *exoc-7;exoc-8* double mutants.

*rab-10(dx-2)* mutation induces the formation of large GFP::RAB-5-positive early endosomes [33]. To verify that that RAB-5 accumulates in *exoc-7*, *exoc-8* and *exoc-7;exoc-8* mutant worms that are treated with *rab-10* RNAi, GFP::RAB-5 labeling in the early endosomes was quantified in the intestinal cells. Consistent with the results for hTAC, the GFP::RAB-5 signal increased in *exoc-8* and *exoc-7;exoc-8* mutants subjected to *rab-10* RNAi (Figure 7B). This result, together with the observed size increase for RAB-7-positive vacuoles (Figure 5) suggests that *rab-10*, *exoc-7* and *exoc-8* cooperate in basolateral endocytic recycling in the *C. elegans* intestine.

GFP::RME-1 labels the basolateral recycling endosomes and there is a clear reduction in RME-1-positive intracellular membranes in *rab-10* defective intestinal epithelial cells implying a block in transport from early endosomes to recycling endosomes [33]. The *rab-10* RNAi-induced loss of the RME-1-positive basal recycling endosomes was suppressed in *exoc-7*, *exoc-8* single and *exoc-7;exoc-8* double mutants (Figure 7C). The observed suppression is likely to be due to a transport block of GFP::RME-1 in the recycling endosomes. This pool of GFP::RME-1 is likely to represent proteins that had managed to escape the effect of *rab-10* RNAi form early endosome export. In the absence of a fully functional route back to the plasma membrane in exocyst defective cells, accumulation in the recycling endosomes is observed.

The analysis of the signal for, the apical recycling endosomes marker GFP::RAB-11 revealed that the apical recycling is not affected by *exoc-7*, *exoc-8* or *exoc-7;exoc-8* (Figure S7C). Furthermore, *exoc-7;exoc-*8 double mutant worms exposed to apical Rhodamin-labeled dextran (Figure S8A) and FM 4-64 (Figure S8B) by feeding showed normal uptake that ultimately led to colocalization of dyes and the autofluorescent gut granules. This suggests that *exoc-7* and *exoc-8* do not perform an essential function in apical recycling in the *C. elegans* intestine.

## Discussion

In *C. elegans*, a number of behavioral assays have been employed to identify mutants with defective cilia, which are thin membrane protrusions that have sensory functions [25], [37]. Here we report that the exocyst subunit *exoc-7*, *exoc-8* single and *exoc-7;exoc-8* double mutant worms show differential behavioral defects that resemble cilia mutant phenotypes (Figure 2). However, the observed phenotypes are unlikely to be caused by defects on overall cilia structure as no obvious defects were observed in sensory neurons (Figure S4). While an extensive literature describes the molecular components involved in the transport within the cilium [38], little is known about the contribution of *exoc-7* and *exoc-8* in the cilia function. Our results suggest that the intracellular trafficking to cilia is compromised in the absence of fully functional *exoc-7* and/or *exoc-8* and that this may cause the observed sensory defects. Previous results show that exocyst subunit Sec10 is important for ciliogenesis in mammalian epithelial cells [39], [40]. The molecular details underlying the behavioral phenotypes observed for *exoc* mutants is currently unclear. It is possible that in these mutants the transport of a subset of plasma membrane components required for full cilia function is affected. Clearly, additional experiments on the exocyst function in *C. elegans* cilia are needed to clarify this issue in the future. The *exoc-7;exoc-8* double mutants displayed more severe sensory phenotypes in some assays (chemo- and thermosensation and growth rate), while significantly milder or no phenotypes were observed in other assays (movement, mechanosensation and life span). This may indicate differential roles for these exocyst subunits in different neuronal cell types in *C. elegans*.

The present and other studies indicate that the exocyst subunits are broadly expressed in *C. elegans* (Figures 1 and S1) [24]. Furthermore, previous studies in other model systems have shown a central role for the exocyst in cell polarity generation and maintenance during development [2]–[4], [13]. The molecular mechanisms that govern exocyst function are poorly understood. However, it is clear that the molecular interactions with small GTPases provide means to modify exocyst function [2], [3], [13]. Our epistatis analysis suggests that two small GTPase *ral-1* and *rab-8* act in the same signaling pathway with *exoc-8* in the regulation of chemosensation behavior (Figure 3). Furthermore, eleven genes were identified in an RNAi screen for synthetic lethality in combination with mutations in *exoc-7*, *exoc-8* and *exoc-7;exoc-8*. In the case of *arl-1*, *rheb-1* and *rab-37*, RNAi treatment resulted in a similar Cu^2+^-hypersensitive phenotype that was observed for *exoc-7* and *exoc-8* mutant animals. However, animals subjected to RNAi for *let-60* became apparently insensitive to Cu^2+^ (Figure S6). These results suggest that the exocyst complex acts in combination with various small GTPases to regulate metal avoidance behavior. According to the current functional information on the small GTPases identified in our screen, it is unlikely that they all affect *exoc-7* and *exoc-8* functions through identical mechanisms. The *exoc-7* mutant displayed less severe phenotypes in the synthetic lethality screen, possibly due to the residual transcription of the 5′ end of the *exoc-7* gene in the *exoc-7(ok2006)* mutant animals (Figure S2). However, based on enhanced phenotypes observed in RNAi screen for *exoc-7* single and *exoc-7;exoc-8* double mutants, the *exoc-7(0k2006)* mutant allele can still be considered as a hypomorphic, reduction-of-function mutant (Figure 4).

The screen identified several genes that have been previously implicated in exocyst function regulation in other model systems. These include *cdc-42*, *rab-8*, *rab-10* and *rap-1*. CDC-42 is a Rho GTPase that controls polarity of both individual cells and developing embryos and is known to interact with Sec3p in yeast [41]. Furthermore, *exoc-7* orthologues interact with the CDC-42 homologue TC10 in mammalian cells [42]. The *rab-8* has been reported to participate in many of the pathways associated with the exocyst complex: basolateral membrane transport [43], plasma membrane remodeling, insulin-dependent Glut4 traffic [34], [44] and ciliogenesis [39]. Rab10 has been reported to associate with primary cilium and with the basal body of nascent cilia of renal epithelia. Itcoimmunoprecipitates with exocyst protein complex subunit Sec8, suggesting coexistence within the same protein complex [34]. The *rap-1* has been shown to coordinate the RAL-1/exocyst pathway in mediating hypodermal cell movement and elongation during embryonic development in *C. elegans* [45]. In addition, silencing of *exoc-8* caused lethality in *rap-1* mutant animals [45]. The fact that our screen identified several known exocyst interacting genes, suggests that the screen has targeted the exocyst function.

In the RNAi screen, several novel potential exocyst regulators were indentified. The *let-60* gene acts genetically downstream of *let-23* with respect to vulva development and upstream of the MAPK pathway with respect to chemotaxis [46], [47]. The *rab-1*, *rab-6.2*, *rab-28* and *rab-37* have all been shown in other model systems to regulate intracellular membrane trafficking. In the case of RAB-37, the protein is involved in secretory granule exocytosis in mammalian cells [48]. The membrane traffic step regulated by RAB-28 activity is unknown [12]. However, our findings suggest that RAB-28 may be functionally linked to exocyst regulation. *rheb-1*, an upstream activator of TOR signaling is required for normal growth rates, lifespan, body size, osmoregulation, reproduction, and locomotion [49]. Furthermore, *rheb-1* has also been shown to mediate intermittent fasting-induced longevity in *C. elegans* [50]. In yeast *Schizosaccharomyces pombe*, a Rab6 homologue Ryh1 can activate TOR signaling [51]. Our results linking *rheb-1* to defects in exocyst subunits raise the possibility that exocyst function is directly or indirectly linked to TOR signaling regulation in *C. elegans*. Our results implicate a general requirement for small GTPases in regulating exocyst function in various physiological events including cell polarity, embryogenesis, intracellular membrane trafficking, endocytic recycling, chemosensation and growth.

Previous studies suggest that the exocyst serves as an effector for Rab10 in cultured mammalian epithelial cells [34]. An RNAi screen in *C. elegans* for defective transport vesicle adaptor complex 1 component AP-1 showed that *rab-10* is important for von Willebrand factor secretion [52]. Here we present the first *in vivo* studies in a multicellular model organism showing that *exoc-7* and *exoc-8* are functionally linked to the small GTPase *rab-10*. When combined with *exoc-8* and *exoc-7;exoc-8* mutants, down-regulation of *rab-10* expression by RNAi resulted in enlarged endosomal membrane compartments in the intestinal epithelial cells (Figure 5). Unlike *rab-10* mutants that display an obvious endocytic recycling phenotype in the intestinal cells [33], we did not detect recycling defects in *exoc-7* or *exoc-8* deletion alleles alone. However, an enhanced phenotype was observed when *rab-10*, *exoc-7* and *exoc-8* were all affected.

The RAB-10 expression is up-regulated when combined with *exoc-8* and *exoc-7;exoc-8* mutants in *C. elegans* (Figure 6). It appears that defects in exocyst complex subunits *exoc-7* and *exoc-8* can directly or indirectly affect the protein expression of small GTPase RAB-10. This result supports the recently identified close functional relationship between Rab10 and the exocyst complex in mammalian epithelial cells [34]. Our results suggest that endocytic recycling and membrane transport are mediated by *rab-10* and exocyst subunits located in intestinal cells in *C. elegans*. The exocyst complex, originally associated with basolateral membrane transport from the trans-Golgi network of polarized mammalian cells [8], [9], [53] is increasingly acknowledged as an important regulator of membrane recycling from the endosomal compartment to the plasma membrane. RAB-10 is thought to mediate cargo recycling from early endosomes to recycling endosomes in *C. elegans* intestine [33]. Such a hypothesis is consistent with our observation that *rab-10* mutant animals display abnormally large early endosomes and lack recycling endosomes in intestinal cells.

To gain a deeper understanding of how loss of *exoc-8* and *exoc-7;exoc-8* functionally enhances *rab-10* phenotype, the localization of a set of GFP tagged intracellular marker proteins was analyzed in intestinal cells in *exoc-7* and *exoc-8* mutant animals subjected to *rab-10* RNAi. There was no obvious difference in the accumulation of the endocytic marker proteins (hTAC and hTfR) in *exoc-7*, *exoc-8* single and *exoc-7;exoc-8* double mutants compared to wild type animals (Figure 7 and Figure S7). This indicates that the internalization step of endocytosis is not impaired. However, the accumulation of endocytic tracers increased significantly when *exoc-8* and *exoc-7;exoc-8* double mutant animals were subjected to *rab-10* RNAi.

The trafficking of cargo from the cell membrane to the lysosome requires the activity of *rab-5*. The average intensity of GFP::RAB-5-positive early endosomes in the absence of *rab-10* increased in *exoc-7;exoc-8* background. This suggests that a block in cargo export from early endosomes is intensified when *rab-10* down-regulation is combined with *exoc-8* or *exoc-7;exoc-8*. At the same time it was observed that *rab-10* RNAi caused a clear reduction in the number of GFP::RME-1-positive basolateral recycling endosomes. However, when *rab-10* was down-regulated in *exoc-8* and *exoc-7;exoc-8* mutant animals, the GFP::RME-1 signal was restored to normal level. It is likely that the reversal of RME-1 protein accumulation in *rab-10* RNAi treated *exoc-8* and *exoc-7;exoc-8* mutant animals reveals a functional role for the exocyst in the transport from the recycling endosomes to the plasma membrane. It is likely that in the absence of a fully functional exocyst complex the pool of GFP::RME-1 that had managed to escape from the early endosomes accumulated to recycling endosomes when no functional recycling route to the cell surface existed anymore.

Currently, we cannot rule out that *exoc-7* and *exoc-8* also participate in cargo transport in the apical surface of *C. elegans* intestine. So far, our attempts to identify apical recycling defects have not been successful. However, no endocytic defects were observed in *exoc-7* and *exoc-8* mutants when worms were fed with FM4-64 and rhodamine-labeled dextran which can be taken up by endocytosis from the lumen of intestine. In support of this hypothesis, we also observed that there is no signal intensity or distribution difference of GFP::RAB-11-positive apical recycling endosome in *exoc-7* and *exoc-8* mutants compared to wild type.

We have identified potential novel regulators of exocyst function and established a novel link between EXOC-7, EXOC-8 and RAB-10 signaling in the regulation of endocytic recycling in *C. elegans* epithelia intestine cells. The exocyst complex is a highly conserved protein complex and thus our studies on *C. elegans* exocyst are likely to be applicable to the understanding of the exocyst complex function in other systems.

## Materials and Methods

### *C. elegans* strains

Strains used in this study were: N2(wild type), *rrf-3*(*pk1426*), *exoc-7*(*ok2006*), *exoc-8*(*ok2523*), *ral-1*(*tm2140*), *unc-10(e102)*, *che-3(e1124)*, OH3191 otIs3[P*gcy-7*::GFP], OH3192 ntIs1[P*gcy-5*::GFP], PY1322 oyIs18[P*gcy-8*::GFP], GJ814 gjIs230[P*gpa-15*::GFP; P*elt-2*::GFP], OR1116 odIs42[P*glr-1*::RFP::RAB-10];odIs22[P*glr-1*::LIN-10::GFP], RT533 pwIs214[P*rab-10*::GFP::RAB-10], RT393 pwIs112[P*vha-6*::hTAC::GFP], RT1970 pwIs90[P*vha-6*::hTfR::GFP], RT327 pwIs72[P*vha-6*::GFP::RAB-5], RT476 pwIs170[P*vha-6*::GFP::RAB-7], RT348 pwIs87[P*vha-6*::GFP::RME-1], RT311 pwIs69[P*vha-6*::GFP::RAB-11]. All strains were maintained utilizing standard methods [54]. Other strains of different mutant background were made by crossing are listed as follows:

*exoc-7*(*ok2006*);*exoc-8*(*ok2523*),
*exoc-7*(*ok2006*);*rrf-3*(*pk1426*),
*exoc-8*(*ok2523*);*rrf-3*(*pk1426*),
*exoc-7*(*ok2006*);*exoc-8*(*ok2523*);*rrf-3*(*pk1426*),
*exoc-7*(*ok2006*);*ral-1*(*tm2140*),
*exoc-8*(*ok2523*);*ral-1*(*tm2140*),
*exoc-7*(*ok2006*);*exoc-8*(*ok2523*);*ral-1*(*tm2140*),
*exoc-7*(*ok2006*);otIs3[P*gcy-7*::GFP],
*exoc-8*(*ok2523*);otIs3[P*gcy-7*::GFP],
*exoc-7*(*ok2006*);*exoc-8*(*ok2523*);otIs3[P*gcy-7*::GFP],
*exoc-7*(*ok2006*);ntIs1[P*gcy-5*::GFP],
*exoc-8*(*ok2523*);ntIs1[P*gcy-5*::GFP],
*exoc-7*(*ok2006*);*exoc-8*(*ok2523*);ntIs1[P*gcy-5*::GFP],
*exoc-7*(*ok2006*);oyIs18[P*gcy-8*::GFP],
*exoc-8*(*ok2523*);oyIs18[P*gcy-8*::GFP],
*exoc-7*(*ok2006*);*exoc-8*(*ok2523*);oyIs18[P*gcy-8*::GFP],
*exoc-7*(*ok2006*);gjIs230[P*gpa-15*::GFP; P*elt-2*::GFP],
*exoc-8*(*ok2523*);gjIs230[P*gpa-15*::GFP; P*elt-2*::GFP],
*exoc-7*(*ok2006*);*exoc-8*(*ok2523*);gjIs230[P*gpa-15*::GFP; P*elt-2*::GFP],
*exoc-7*(*ok2006*);odIs42[P*glr-1*::RFP::RAB-10];odIs22[P*glr-1*::LIN-10::GFP],
*exoc-8*(*ok2523*);odIs42[P*glr-1*::RFP::RAB-10];odIs22[P*glr-1*::LIN-10::GFP],
*exoc-7*(*ok2006*);*exoc-8*(*ok2523*);odIs42[P*glr-1*::RFP::RAB-10];odIs22[P*glr-1*::LIN-10::GFP],
*exoc-7*(*ok2006*);pwIs214[P*rab-10*::GFP::RAB-10],
*exoc-8*(*ok2523*);pwIs214[P*rab-10*::GFP::RAB-10],
*exoc-7*(*ok2006*);*exoc-8*(*ok2523*);pwIs214[P*rab-10*::GFP::RAB-10],
*exoc-7*(*ok2006*);pwIs112[P*vha-6*::hTAC::GFP],
*exoc-8*(*ok2523*);pwIs112[P*vha-6*::hTAC::GFP],
*exoc-7*(*ok2006*);*exoc-8*(*ok2523*);pwIs112[P*vha-6*::hTAC::GFP],
*exoc-7*(*ok2006*);pwIs90[P*vha-6*::hTfR::GFP],
*exoc-8*(*ok2523*);pwIs90[P*vha-6*::hTfR::GFP],
*exoc-7*(*ok2006*);*exoc-8*(*ok2523*);pwIs90[P*vha-6*::hTfR::GFP],
*exoc-7*(*ok2006*);pwIs72[P*vha-6*::GFP::RAB-5],
*exoc-8*(*ok2523*);pwIs72[P*vha-6*::GFP::RAB-5],
*exoc-7*(*ok2006*);*exoc-8*(*ok2523*);pwIs72[P*vha-6*::GFP::RAB-5],
*exoc-7*(*ok2006*);pwIs170[P*vha-6*::GFP::RAB-7],
*exoc-8*(*ok2523*);pwIs170[P*vha-6*::GFP::RAB-7],
*exoc-7*(*ok2006*);*exoc-8*(*ok2523*);pwIs170[P*vha-6*::GFP::RAB-7],
*exoc-7*(*ok2006*);pwIs87[P*vha-6*::GFP::RME-1],
*exoc-8*(*ok2523*);pwIs87[P*vha-6*::GFP::RME-1],
*exoc-7*(*ok2006*);*exoc-8*(*ok2523*);pwIs87[P*vha-6*::GFP::RME-1],
*exoc-7*(*ok2006*);pwIs69[P*vha-6*::GFP::RAB-11],
*exoc-8*(*ok2523*);pwIs69[P*vha-6*:: GFP::RAB-11],
*exoc-7*(*ok2006*);*exoc-8*(*ok2523*);pwIs69[P*vha-6*:: GFP::RAB-11].

### Ciliary phenotypic analyses

The locomotion assay was done as described by Koelle and Horvitz [55]. The body bends per minute were counted of worms moving forward continuously at 20°C on NGM plates spread with a thin layer of freshly grown OP50 bacteria.

Chemosensation to Cu^2+^ was analyzed with small modifications using a previously described assay [56], [57]. Briefly, in total 200–400 animals were placed on one side of a Cu^2+^ (100 mM) line barrier on a 9 cm assay plate, and sodium azide (NaN_3_) was spotted on the other side to immobilize worms. After 60 min, the number of animals on each side was scored. The index represents the fraction of animals that crossed Cu^2+^ line of the total number of animals. Data from at least three independent experiments were used for quantification analysis.

Nose touch was assayed by placing an eyebrow on the surface of the NGM plates spread with a thin layer of freshly grown OP50 bacteria in front of the forward-moving animal. Upon contact with the tip of the nose the worms initiated immediately backward locomotion. The number of body bends during the backward movement were quantified before the animals changed the direction of movement.

The thermosensation assay was performed on NGM plates pre-heated at 35°C using at least 50 animals for each genotype per experiment. Adults were incubated at 35°C, and were observed every hour by response to touch until all animals became immobile. Data from at least three independent experiments were used for quantification analysis.

For the growth rate assay the time required for the development from newly laid eggs to adults was scored on NGM plates seeded with OP50 bacteria.

Aldicarb and levamisole resistance assays were carried out according to the description of Lackner *et al* [58]. In each experiment, 25–30 worms were placed on drug containing plates and touched every 10 minutes. Worms that failed to respond at all to a harsh touch were classified as paralyzed. Experiments were repeated three times.

### Dye staining

A stock solution containing 20 mg/ml DiI (1,1′-dioctadecyl-3,3,3′,3′,-tetramethylindo-carbocyanine perchlorate, Aldrich) in dimethylformamide was stored at −20°C. A 1.4 µl aliquote of the stock solution was mixed with 700 µl M9 buffer (22 mM KH_2_PO_4_, 42 mM Na_2_HPO_4_, 86 mM NaCl, 1 mM MgSO_4_) to give a final working concentration of 40 µg/ml. Tubes were protected from light with aluminum foil and incubations were carried out at room temperature. Similarly, Rhodamine-dextran (Sigma, St. Louis, MO) and FM4-64 were diluted with egg salt (118 mM NaCl, 48 mM KCl, 2 mM MgCl_2_, 2 mM CaCl_2_, 10 mM HEPES, pH 7.4). Animals were washed off from the growth plate and rinsed twice with M9 buffer then suspended in the dye solution. After shaking for 2 h, the stained animals were washed twice with M9 buffer and subjected to imaging. Experiments were repeated three times.

### RNAi screen

The RNAi screen was performed by feeding bacterial clones on 6-well NGM plates containing 1 mM isopropylthiogalactoside (IPTG) to *rrf-3*, *rrf-3;exoc-7*, *rrf-3;exoc-8* and *rrf-3;exoc-7;exoc-8* mutant animals that had been synchronized to L1 stage [59]. The animals were allowed to grow for 3 days before observing the phenotype. All 41 small GTPase RNAi clones were tested in duplicates and the candidate genes were confirmed in three more independent experiments and subsequently retested in N2 background with at least two repetitions.

### DNA constructs

Transcriptional fusion was created for *exoc-7* by using PCR to amplify 2954 bp intergenic promoter sequences together with the first 72 amino acids of the coding region, followed by introduction of this sequence into the GFP vector pPD118.25 (Andrew Fire Lab Vector Kit, L3786, Addgene) in place of the *let-858* promoter. For the *sec-6* transcriptional fusion reporter, 2065 bp intergenic promoter sequences together with the first 9 amino acids were used.

### Germline transformation

Germline transformation was performed by a standard microinjection method [60], [61] at a concentration of 10 ng/µl for the DNA to be tested and 100 ng/µl *rol*-*6* marker (pRF4).

### RNA isolation and quantitative RT-PCR

Animals subjected to RNAi were collected and washed 3 times with M9. After removing the supernatant, worm pellets were stored at −80°C until RNA isolation. The total RNA was extracted from approximately 1,000 animals for each treatment using the Total RNA Isolation kit (Macherey-Nagel, Germany) and the first-strand cDNA was synthesized using the Maxima First-Strand Synthesis Kit for RT-qPCR (Fermentas). SYBR Green Real Time Quantitative PCR was carried out using the LightCycler® 480 Real-Time PCR System (Roche). In each qRT-PCR assay we used 3 biological replicates and experiments are repeated two times. The α-tubulin was used for normalization. Primer sequences are available in Table S3.

### Confocal microscopy

All static microscope images were acquired using Leica TCS SP5 Laser scanning confocal microscopy with 20×glycerol objective. Confocal settings used for image capture were held constant for same maker strains in experiments. Images were quantified and analyzed using ImageJ software (NIH). The worm fluorescence imaging and quantification were done as previously described [62]. The average pixel intensity in wild type worms was set to an arbitrary fluorescence unit (A.U.) of 1.0 to enable comparison with other strains.

### Western blot assay

For analysis of GFP levels, worms from 9 cm EPM plates were harvested by centrifugation at 4000 rpm for 3 min. After washing with M9 buffer three times, worms were directly boiled for 10 min in 300 µl 2% SDS buffer containing protease inhibitors. Each sample was centrifuged for 5 min at 13,000 rpm and the protein concentration was determined with protein assay kit (Thermo Scientific). Identical amounts of total protein was subjected to SDS-PAGE and transferred to nitrocellulose membrane (D106089, BIO-RAD) following standard procedures. The GFP-tagged proteins were detected with anti-GFP antibody (98028, BD). The α-tubulin was used for normalization.

### Data analysis

Data analysis was conducted using IGOR Pro (Wavemetrics) or EXCEL (Microsoft) software. Averaged results were presented as the mean value ± S.E.M.. Statistical significance was evaluated using Student's t test. Asterisks denote statistical significance as compared to controls, with a P value less than 0.05 (*), 0.01 (**) and 0.001 (***).

## Supporting Information

Figure S1 **Confocal image of adult hermaphrodites expressing P** ***sec-6*** **::GFP under the 2065 bp promoter (Ex[P** ***sec-6::*** **GFP;** ***pRF4*** **]).** Left is anterior. Scale bar, 100 µm.
(TIF)

Figure S2 **Characterization of the** ***exoc-7*** **and** ***exoc-8*** **mutations.** (A) A cartoon displaying the deletion regions in *exoc-7(ok2006)* and *exoc-8(ok2523)* alleles. The red and green arrows indicate the oligonucleotide pairs upstream and downstream, respectively, used for RT-PCR to detect mRNA expressed from these loci. (B) Agarose gel analysis for RT-PCR of *exoc-7(ok2006)* and *exoc-8(ok2523)* worms. M, molecular weight marker. For *exoc-7*, the expected sizes for the amplified fragments are 437 bp and 475 bp for before and after deletion region. For *exoc-8*, the expected sizes for the amplified fragments are 498 bp and 712 bp for before and after deletion region.
(TIF)

Figure S3 (A) *exoc-7*, *exoc-8* and *exoc-7;exoc-8* double mutant worms show hypersensitivity to 100 mM Cd^2+^. The assay setup was the same as described in Figure 2B. (B) *exoc-7*, *exoc-8* and *exoc-7;exoc-8* double mutant worms show hypersensitivity to Cu^2+^ at different concentrations (10 mM, 50 mM and 100 mM). (C) The *unc-10(e102)* mutany animals (n = 22) have a more severe uncoordinated movement defect than the *exoc-8* (n = 27) worms. (D) Quantification of the Cu^2+^-sensitivity for *unc-10* and *exoc-8* worms.
(TIF)

Figure S4 ***exoc-7*** **,** ***exoc-8*** **and** ***exoc-7;exoc-8*** **double mutant worms show no obvious morphological defects in cilia structure by DiI staining.** For a cilia defect, the dynein heavy chain mutant *che-3(e1124)* was used as a control. In addition, no apparent morphological defects are observed in Cu^2+^ sensory neurons ASEL/ASER, ADL, ASH and the thermosensory neuron AFD.
(TIF)

Figure S5 **qRT-PCR quantification of the RNA silencing efficiency for a set of the candidate genes in** ***rrf-3*** **worms.** The mRNA levels of controls were set as arbitrary unit 1.
(TIF)

Figure S6 **Cu^2+^ sensitivity assay indentified that** ***arl-1*** **,** ***rheb-1*** **and** ***rab-37*** **RNAi worms show hypersensitivity to copper ions, whereas** ***let-60*** **RNAi worms are insensitive.**
(TIF)

Figure S7 ***rab-10*** **RNAi does not affect the signal for hTfR::GFP and GFP::RAB-11 in** ***exoc-7*** **,** ***exoc-8*** **and** ***exoc-7;exoc-8*** **double mutant worms.** For hTfR: WT n = 27, *exoc-7* n = 23, *exoc-8* n = 25, *exoc-7;exoc-8* n = 31, WT treated *rab-10* RNAi n = 27, *exoc-7* treated *rab-10* RNAi n = 22, *exoc-8* treated *rab-10* RNAi n = 28, *exoc-7;exoc-8* treated *rab-10* RNAi n = 26. For RAB-11: WT n = 23, *exoc-7* n = 22, *exoc-8* n = 30, *exoc-7;exoc-8* n = 27, WT treated *rab-10* RNAi n = 25, *exoc-7* treated *rab-10* RNAi n = 24, *exoc-8* treated *rab-10* RNAi n = 26, *exoc-7;exoc-8* treated *rab-10* RNAi n = 22.
(TIF)

Figure S8 ***exoc-7*** **,** ***exoc-8*** **and** ***exoc-7;exoc-8*** **double mutant worms show no obvious defects in the uptake of rhodamine-dextran or FM 4-64 from the apical surface of intestinal cells.**
(TIF)

Table S1 **Quantification of pharynx pumping rate, brood size and life span.**
(DOCX)

Table S2 **Description of the candidate genes identified in the screen.**
(DOCX)

Table S3 **Sequences of the oligonucleotides used for qRT-PCR.**
(DOCX)
