# Correction: Exocyst Subunits Exo70 and Exo84 Cooperate with Small GTPases to Regulate Behavior and Endocytic Trafficking in *C. elegans*

**DOI:** 10.1371/annotation/5c145402-1089-4764-a204-e620e15b0e5b

**Published:** 2012-06-07

**Authors:** Yaming Jiu, Congyu Jin, Yanbo Liu, Carina I. Holmberg, Jussi Jäntti

The author Jussi Jäntti has a second affiliation that was not listed. It is: 3. VTT Technical Research Centre of Finland, Espbo, Finland 

