# Supplementary figures and images for: Exocyst Subunits Exo70 and Exo84 Cooperate with Small GTPases to Regulate Behavior and Endocytic Trafficking in C. elegans
Source: PLoS One. 2012 Feb 28;7(2):e32077. doi: 10.1371/journal.pone.0032077 (PMC3289633; doi:10.1371/journal.pone.0032077)

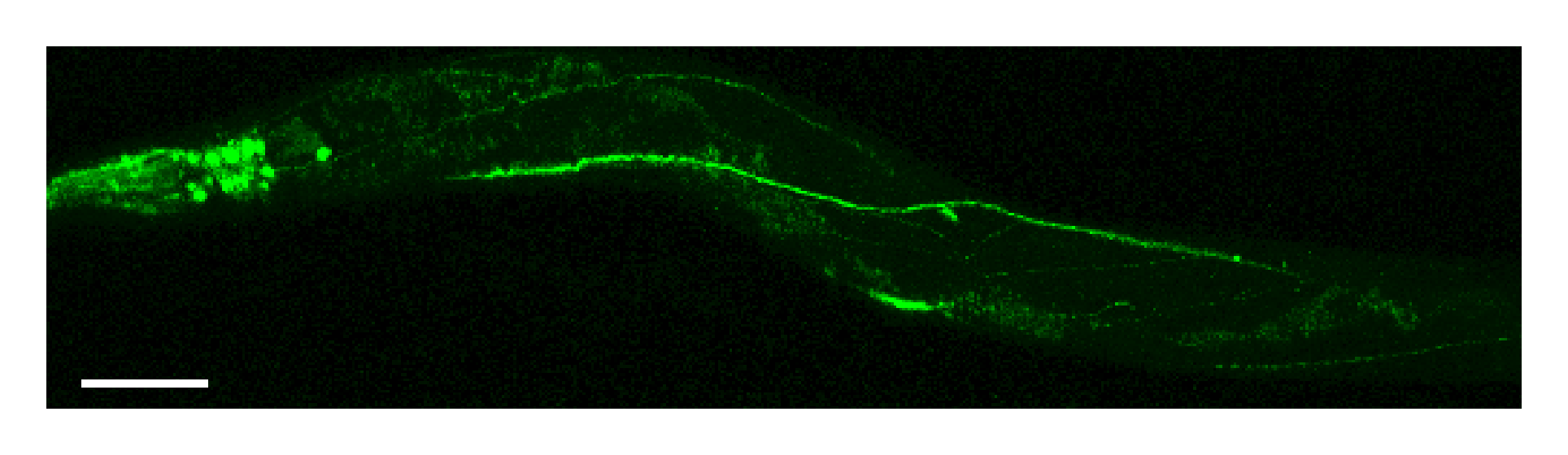

Supplement: Figure S1 — Confocal image of adult hermaphrodites expressing P sec-6 ::GFP under the 2065 bp promoter (Ex[P sec-6:: GFP; pRF4 ]). Left is anterior. Scale bar, 100 µm. (TIF) [file pone.0032077.s001.tif]

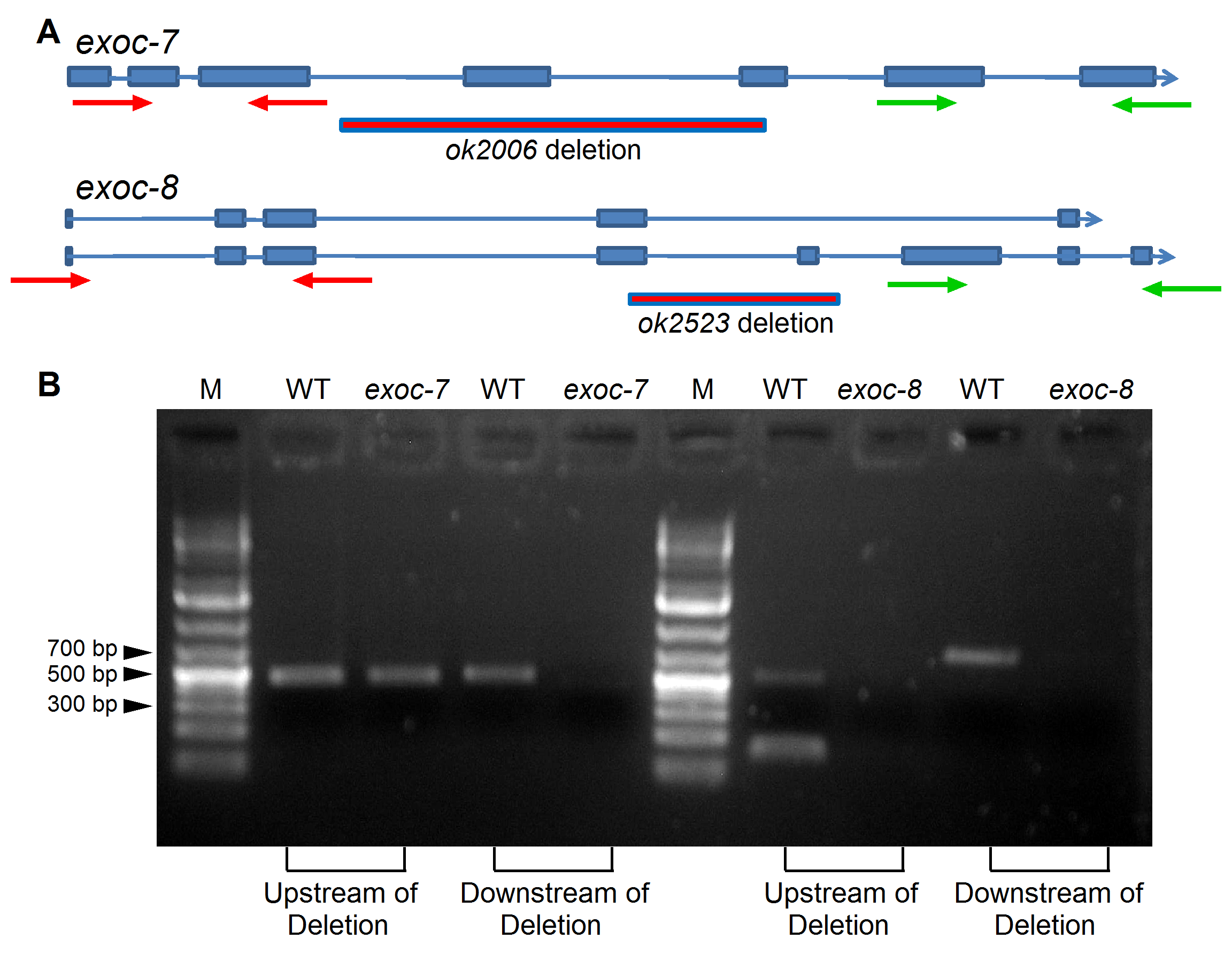

Supplement: Figure S2 — Characterization of the exoc-7 and exoc-8 mutations. (A) A cartoon displaying the deletion regions in exoc-7(ok2006) and exoc-8(ok2523) alleles. The red and green arrows indicate the oligonucleotide pairs upstream and downstream, respectively, used for RT-PCR to detect mRNA expressed from these loci. (B) Agarose gel analysis for RT-PCR of exoc-7(ok2006) and exoc-8(ok2523) worms. M, molecular weight marker. For exoc-7, the expected sizes for the amplified fragments are 437 bp and 475 bp for before and after deletion region. For exoc-8, the expected sizes for the amplified fragments are 498 bp and 712 bp for before and after deletion region. (TIF) [file pone.0032077.s002.tif]

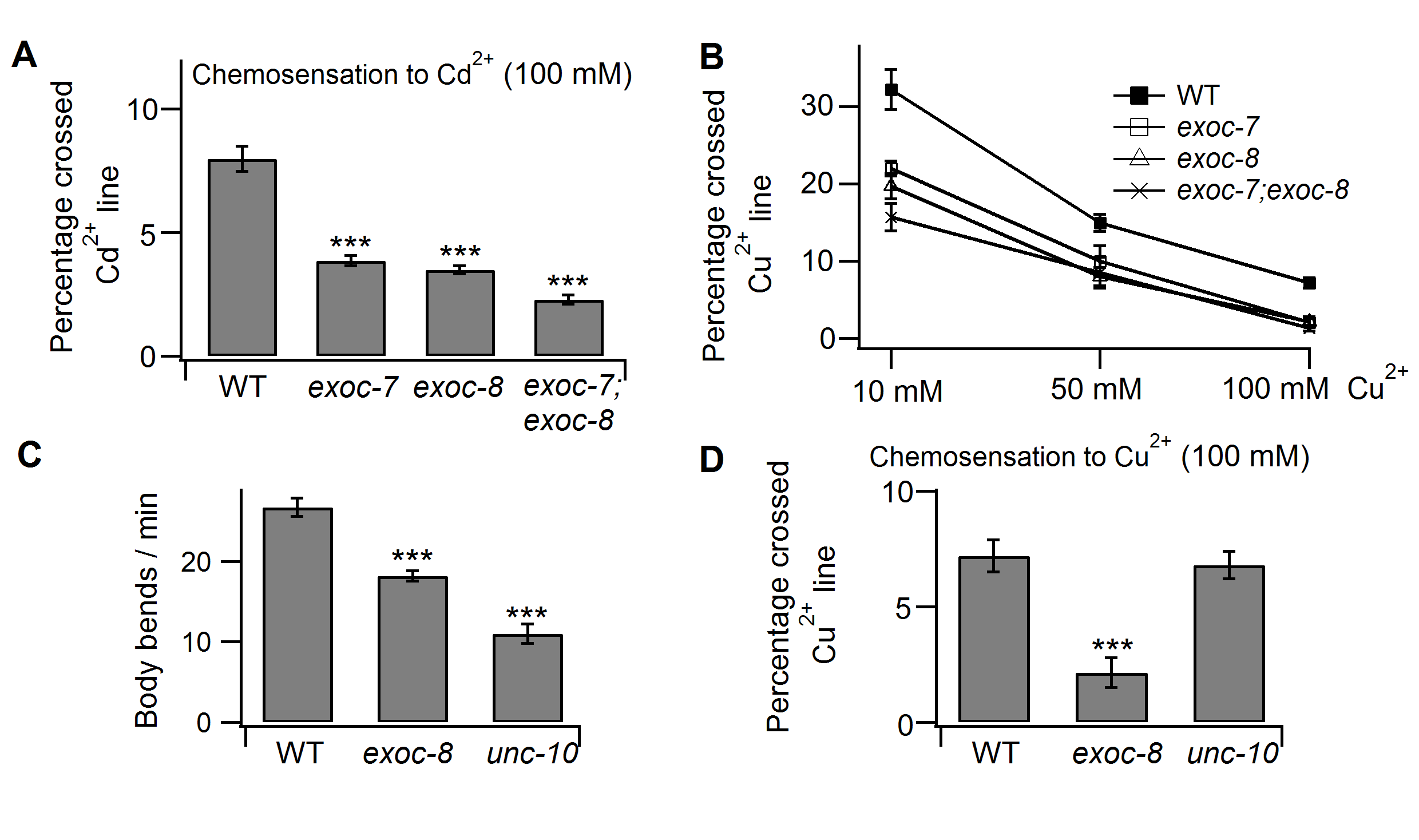

Supplement: Figure S3 — (A) exoc-7, exoc-8 and exoc-7;exoc-8 double mutant worms show hypersensitivity to 100 mM Cd2+. The assay setup was the same as described in Figure 2B. (B) exoc-7, exoc-8 and exoc-7;exoc-8 double mutant worms show hypersensitivity to Cu2+ at different concentrations (10 mM, 50 mM and 100 mM). (C) The unc-10(e102) mutany animals (n = 22) have a more severe uncoordinated movement defect than the exoc-8 (n = 27) worms. (D) Quantification of the Cu2+-sensitivity for unc-10 and exoc-8 worms. (TIF) [file pone.0032077.s003.tif]

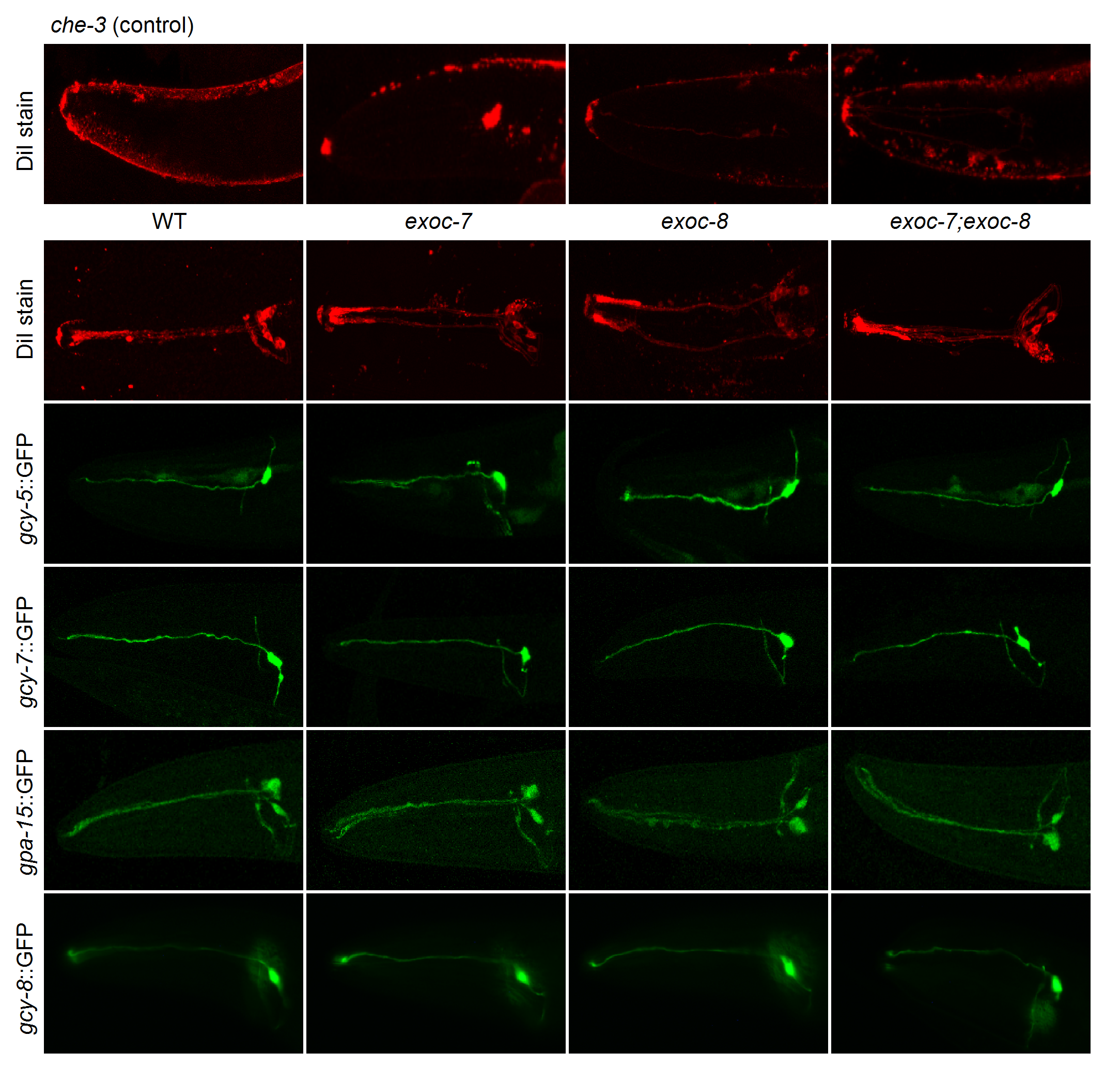

Supplement: Figure S4 — exoc-7 , exoc-8 and exoc-7;exoc-8 double mutant worms show no obvious morphological defects in cilia structure by DiI staining. For a cilia defect, the dynein heavy chain mutant che-3(e1124) was used as a control. In addition, no apparent morphological defects are observed in Cu2+ sensory neurons ASEL/ASER, ADL, ASH and the thermosensory neuron AFD. (TIF) [file pone.0032077.s004.tif]

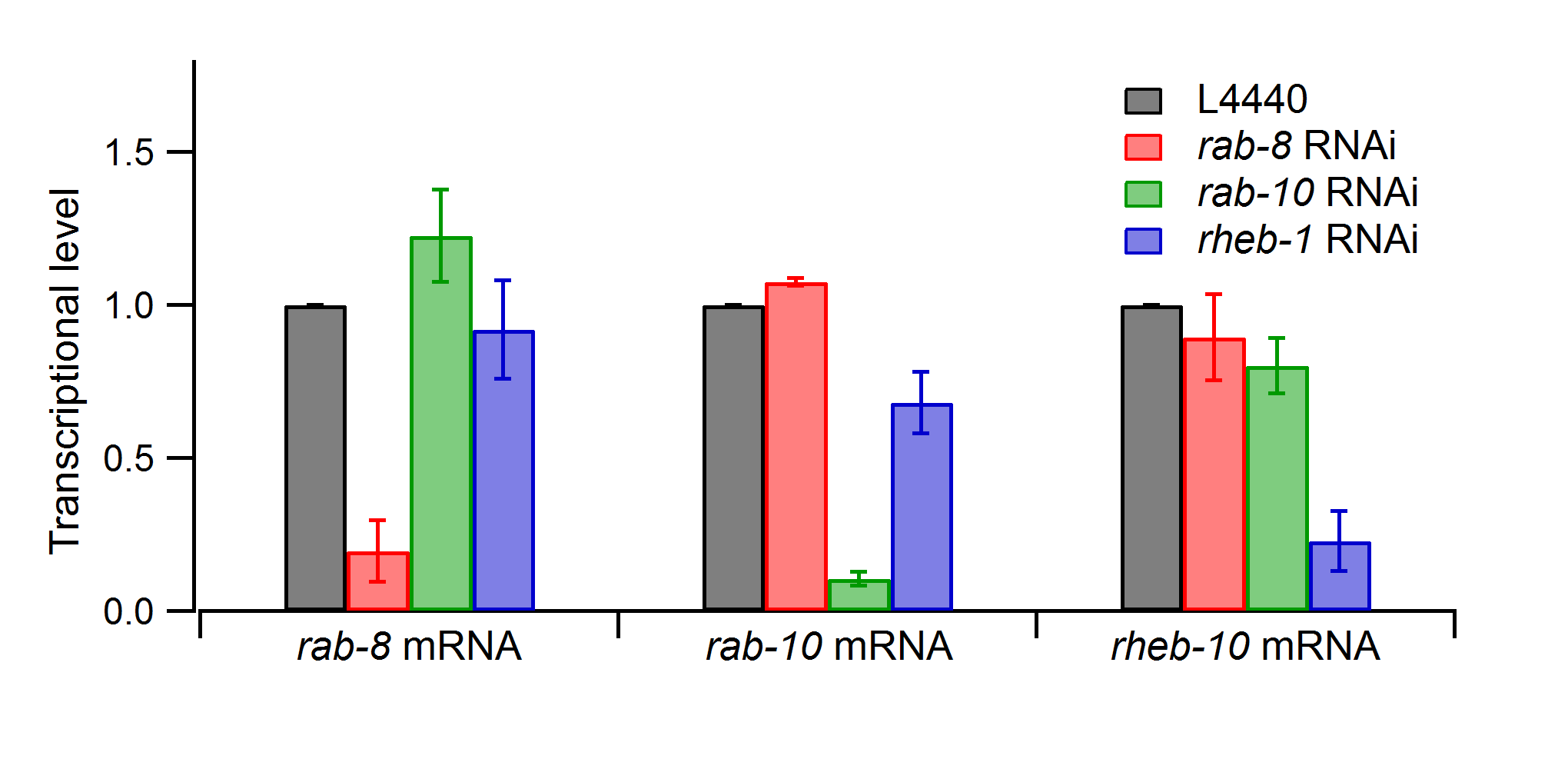

Supplement: Figure S5 — qRT-PCR quantification of the RNA silencing efficiency for a set of the candidate genes in rrf-3 worms. The mRNA levels of controls were set as arbitrary unit 1. (TIF) [file pone.0032077.s005.tif]

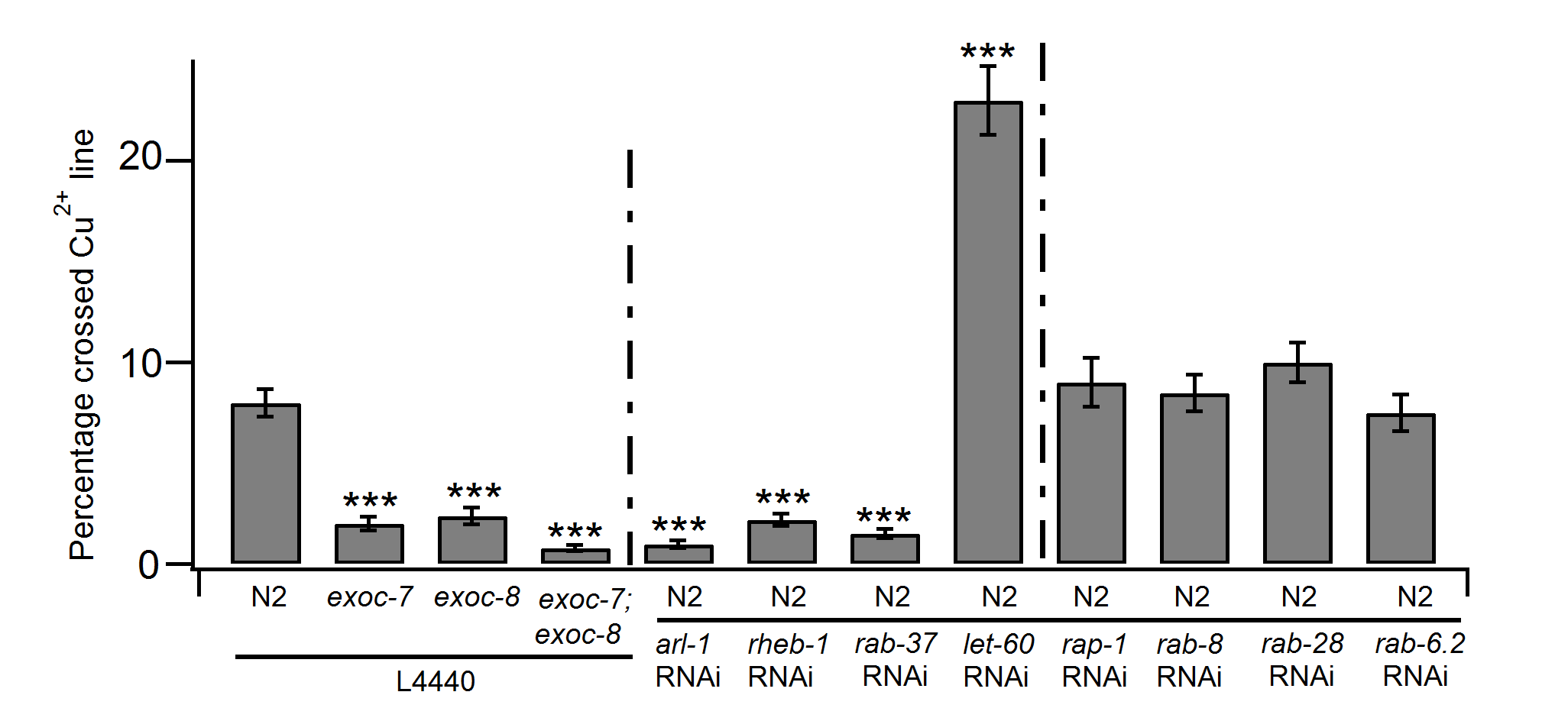

Supplement: Figure S6 — Cu2+ sensitivity assay indentified that arl-1 , rheb-1 and rab-37 RNAi worms show hypersensitivity to copper ions, whereas let-60 RNAi worms are insensitive. (TIF) [file pone.0032077.s006.tif]

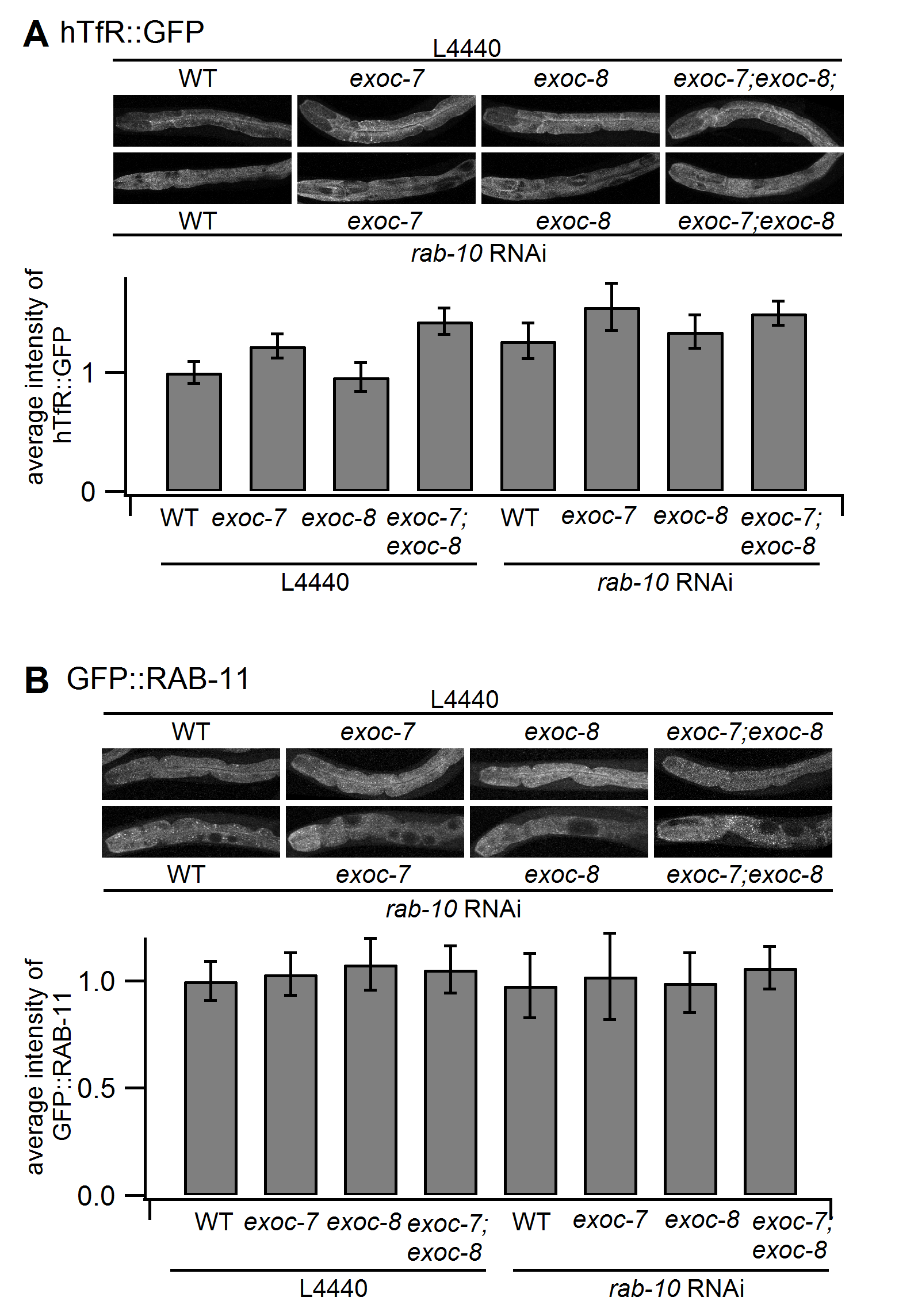

Supplement: Figure S7 — rab-10 RNAi does not affect the signal for hTfR::GFP and GFP::RAB-11 in exoc-7 , exoc-8 and exoc-7;exoc-8 double mutant worms. For hTfR: WT n = 27, exoc-7 n = 23, exoc-8 n = 25, exoc-7;exoc-8 n = 31, WT treated rab-10 RNAi n = 27, exoc-7 treated rab-10 RNAi n = 22, exoc-8 treated rab-10 RNAi n = 28, exoc-7;exoc-8 treated rab-10 RNAi n = 26. For RAB-11: WT n = 23, exoc-7 n = 22, exoc-8 n = 30, exoc-7;exoc-8 n = 27, WT treated rab-10 RNAi n = 25, exoc-7 treated rab-10 RNAi n = 24, exoc-8 treated rab-10 RNAi n = 26, exoc-7;exoc-8 treated rab-10 RNAi n = 22. (TIF) [file pone.0032077.s007.tif]

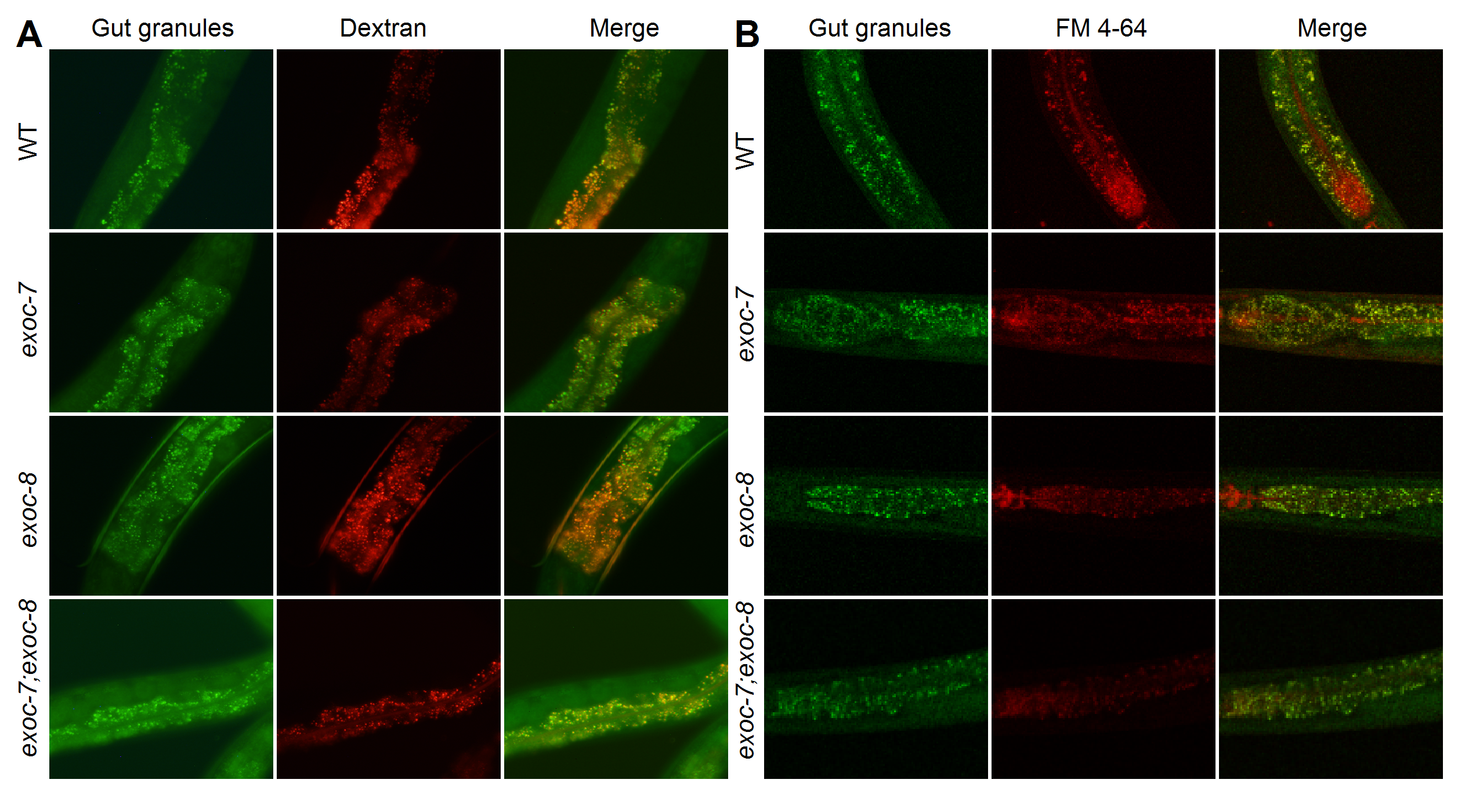

Supplement: Figure S8 — exoc-7 , exoc-8 and exoc-7;exoc-8 double mutant worms show no obvious defects in the uptake of rhodamine-dextran or FM 4-64 from the apical surface of intestinal cells. (TIF) [file pone.0032077.s008.tif]
