# Supplementary material for: Exocyst Subunits Exo70 and Exo84 Cooperate with Small GTPases to Regulate Behavior and Endocytic Trafficking in C. elegans
Source: PLoS One. 2012 Feb 28;7(2):e32077. doi: 10.1371/journal.pone.0032077 (PMC3289633; doi:10.1371/journal.pone.0032077)
Supplement: Table S1 — Quantification of pharynx pumping rate, brood size and life span. (DOCX) [file pone.0032077.s009.docx]

| **Genotype** | **Pumping rate / min** | **Brood size / worm** | **Median Life span (day)** |
| --- | --- | --- | --- |
| **WT** | 232 ± 3 | 252 ± 21 | 16.3 ± 3.7 |
| ***exoc-7*** | 230 ± 5 | 257 ± 22 | 17.1 ± 4.6 |
| ***exoc-8*** | 224 ± 3 | 243 ± 19 | 17.3 ± 4.9 |
| ***exoc-7;exoc-8*** | 226 ± 7 | 235 ± 23 | 16.1 ± 3.5 |
