# Supplementary material for: Exocyst Subunits Exo70 and Exo84 Cooperate with Small GTPases to Regulate Behavior and Endocytic Trafficking in C. elegans
Source: PLoS One. 2012 Feb 28;7(2):e32077. doi: 10.1371/journal.pone.0032077 (PMC3289633; doi:10.1371/journal.pone.0032077)
Supplement: Table S2 — Description of the candidate genes identified in the screen. (DOCX) [file pone.0032077.s010.docx]

| **RNAi clone** | **Gene** | **Domains and description** |
| --- | --- | --- |
| T23H2.5 | *rab-10* | Ras superfamily of small GTPases, required for basolateral endocytic recycling in the intestine, but not in oocytes or coelomocytes |
| F54C9.10 | *arl-1* | GTP-binding ADP-ribosylation factor family |
| R07G3.1 | *cdc-42* | a RHO GTPase, controls polarity of both individual cells and developing embryos by regulating the localization of PAR proteins |
| C27B7.8 | *rap-1* | Ras superfamily of small GTPases |
| ZK792.6 | *let-60* | GTP-binding RAS protooncogene family, required for viability, vulval development, spicule development, germ line meiotic progression, posterior development of the hypodermis, chemotaxis, sex myoblast migration, and muscle membrane extension |
| C39F7.4 | *rab-1* | small Ras-like GTPase, required for intracellular vesicle trafficking |
| W01H2.3 | *rab-37* | Ras GTPase superfamily |
| D1037.4 | *rab-8* | Ras GTPase superfamily, affects ovarian morphology and function, acts downstream of *let-60* with respect to vulval development |
| F54C8.5 | *rheb-1* | orthologous to the mammalian Rheb, involved in the mitochondrial unfolded protein response and required for normal growth rates, body size, osmoregulation, reproduction, and locomotion |
| Y11D7A.4 | *rab-28* | Ras GTPase superfamily, function in endocytosis, membrane fusion, and vesicular trafficking |
| [T25G12.4](http://www.wormbase.org/db/seq/sequence?name=T25G12.4;class=Gene_name) | *rab-6.2* | monomeric Rab GTPase, related to the Drosophila and mammalian Rab6 GTPases, required for intracellular membrane trafficking |
