# Supplementary material for: Exocyst Subunits Exo70 and Exo84 Cooperate with Small GTPases to Regulate Behavior and Endocytic Trafficking in C. elegans
Source: PLoS One. 2012 Feb 28;7(2):e32077. doi: 10.1371/journal.pone.0032077 (PMC3289633; doi:10.1371/journal.pone.0032077)
Supplement: Table S3 — Sequences of the oligonucleotides used for qRT-PCR. (DOCX) [file pone.0032077.s011.docx]

| **oligo name** | **oligo sequence** |
| --- | --- |
| *rab-8*_forward | 5’ TTGGGACACAGCCGGGCAGG 3’ |
| *rab-8*_reverse | 5’ CTCCACGGTCCCGCGACACT 3’ |
| *rab-10*_forward | 5’ ACGAGTTGTGAGCAGAGAACGAGG 3’ |
| *rab-10*_reverse | 5’ CGCGGGATTGCTCGTCAGTGG 3’ |
| *rheb-1*_forward | 5’ ACGCAAAGGGTCGTTCGAGCA 3’ |
| *rheb-1*_reverse | 5’ ACCGTTTGGACGCTCCGTTGG 3’ |
| *exoc-7*_forward _1 | 5’ GACAAGGAATCGAACAAGTT 3’ |
| *exoc-7*_reverse_1 | 5’ GGTTCTTCATCGGTCATAAT 3’ |
| *exoc-7*_forward _2 | 5’ GTACGATGATCCAACACTTG 3’ |
| *exoc-7*_reverse_2 | 5’ AGCAGATGAGAATAGATTTCG 3’ |
| *exoc-8*_forward _1 | 5’ GCTTTCAAATGAGGATGG 3’ |
| *exoc-8*_reverse_1 | 5’ ATACACTTCTGCACTGGCT 3’ |
| *exoc-8*_forward _2 | 5’ TCAAGTACTTCAATGGTGTTC 3’ |
| *exoc-8*_reverse_2 | 5’ AGCAACTTCTTCATCATCCT 3’ |
